# Small area mapping of prostate cancer incidence in New York State (USA) using fully Bayesian hierarchical modelling

**DOI:** 10.1186/1476-072X-3-29

**Published:** 2004-12-08

**Authors:** Glen D Johnson

**Affiliations:** 1 New York State Cancer Registry, New York State Department of Health, Albany, NY, USA; 2 Department of Environmental Health and Toxicology, School of Public Health, University at Albany, Albany, NY, USA

## Abstract

**Background:**

As part of a long-term initiative to improve cancer surveillance in New York State, small area maps of relative risk, expressed as standardized incidence ratios (SIRs), were produced for the most common cancers. This includes prostate cancer, the focus of this paper, since it is the most common non-dermatologic malignancy diagnosed among men and the second leading cause of cancer deaths for men in the United States.

ZIP codes were chosen as mapping units for several reasons, including the need to balance between protecting personal privacy and public demand for fine geographic resolution. Since the population size varies greatly among such small mapping units, hierarchical Bayes spatial modelling was applied in this paper to produce a map of smoothed SIRs. It is further demonstrated how other characteristics of the large sample from the stationary posterior distribution of SIRs can be mapped to investigate various aspects of the statewide spatial pattern of prostate cancer incidence.

**Results:**

Thematic mapping of the median and 95 percentile range of SIRs provided, respectively, a map of spatially smoothed values and the uncertainty associated with these smoothed values. Maps were also produced to identify ZIP codes expressing a 95% probability, in the Bayesian paradigm, of being less than or greater than the null value of 1.

**Conclusion:**

The model behaved as expected since areas that were statistically elevated coincided with areas identified by the spatial scan statistic, plus the relative uncertainty increased as a ZIP code's population decreased, with an exaggerated effect for low population ZIP codes on the edge of the state border.

The overall smoothed pattern, along with identified high and low areas, may reflect difference across the state with respect to socio-demographics and risk factors; however, this is confounded by potential differences in screening and diagnostic follow-up. Nevertheless, the Bayes modelling approach is shown to provide not only smoothed results, but also considerable other information from a large empirical distribution of outcomes associated with each mapping unit.

## Background

Geographic surveillance of chronic disease is central to understanding spatial or spatial-temporal patterns that may help to identify discrepancies in disease burden among different regions or communities. As part of ongoing efforts in New York State to understand spatial patterns of cancer and to help implement cancer prevention and control programs, small area maps of cancer relative risk, expressed as standardized incidence ratios (SIRs), have been produced and shared with the public [1] for the most common anatomical cancer sites.

Prostate cancer, the focus of this paper, was included because it is the most common non-dermatologic malignancy diagnosed among men and the second leading cause of cancer deaths for men in the United States (US) [2]. Although mortality from this disease in the US has statistically significantly decreased at a rate of 2.6% per year from 1990 to 2000 [3], unexplained geographic discrepancies in mortality rates do exist [4]. Furthermore, several treatment options appear to be associated with excellent long-term disease-specific survival for otherwise healthy men with localized disease [5].

**Figure 1:**
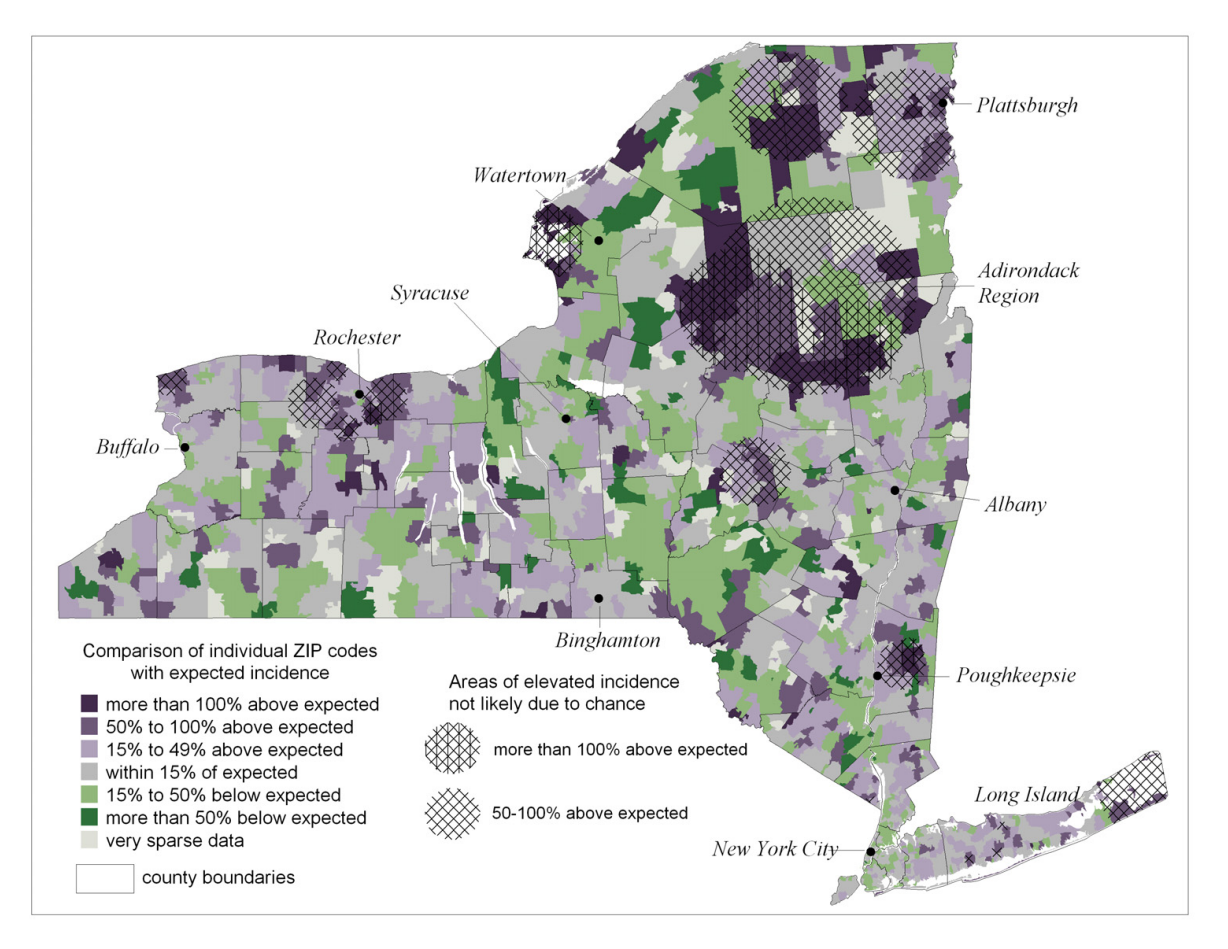
**All stage prostate cancer Incidence by ZIP Code in New York State, 1994–1998**. ZIP code-level ratios of observed incidence to age- and race-adjusted expected incidence, along with significant spatial scan statistic circles that are non-overlapping within specified ranges of standardized incidence ratios, based on reference [1]. Select cities and regions overlaid for reference.

Results for prostate cancer (all stages combined) are reproduced in Figure 1, where ZIP code-level standardized incidence ratios (SIRs) are presented along with results from analyzing these data with the spatial scan statistic [6]. The circles in Figure 1 represent statistically elevated regions based on Poisson likelihood ratios comparing rates inside the circle to those outside the circle. Details of how the scan statistic results were reduced to the circles presented in Figure 1 are found in Boscoe et al [7].

It is well recognized that the stability of population-based statistics like the SIRs in Figure 1 can vary greatly among small geographic areas due to varying population size. Different methods of smoothing have been developed to address this issue, where all are based on the phenomenon that observations close together in space are more likely to share similar properties than those that are far apart [8]. While this positive spatial autocorrelation may be problematic for statistical methods that require independent observations, it can also be embraced to help smooth noisy maps by borrowing strength from neighbors for those mapping units with small populations. Of the different approaches to spatial smoothing, only a few appear to have gained acceptance in spatial epidemiology. Non-parametric approaches include spatial filtering [9,10] and the head-banging algorithm [11], both of which are basically variations on a moving window kernel-type smoother.

The parametric approach of generalized linear modelling [12] treats the observed response, *y*, as a random variable that has arisen from a probability distribution with expectation *θ*. This expectation is modeled, via an appropriate link *g*(·), as a linear function *g*(*θ*) = *α *+ **x'*β ***+ *ε*, for a common value *α*, explanatory covariates **x'*β ***and a random effect *ε *that captures unexplained variation. If the random effect is associated with exchangeable spatial heterogeneity, estimates are smoothed towards a global mean, whereas if the random effect is associated with local spatial autocorrelation, estimates are smoothed towards a local neighborhood mean, which is typically more meaningful in geographic epidemiology. There are different approaches to modelling local spatial dependence, and section 6.3 of Cressie [13] presents several arguments in favor of the conditional autoregressive (CAR) model originally conceived by Besag [14].

Estimation of model parameters can proceed by maximum likelihood [13]; however, the hierarchical nature of generalized linear models lends itself well to Bayesian analysis whereby linear terms in the model are assigned prior distributions that, in turn, have "hyperprior" parameters. Earlier applications employed empirical Bayes methods [15], where hyperparameters are estimated directly from the data. This approach is limited because it assigns a point estimate to the hyperparameter without allowing for variability that may be associated with it, and this variability can be large [16,17].

Fully Bayesian modelling assigns hyperprior distributions to these hyperparameters, so that every parameter of the hierarchical model is allowed to vary over a prior distribution and no single point estimate is used to represent an unknown parameter value. Furthermore, the fully Bayesian approach allows the convolution model that incorporates both a heterogeneous and spatially structured random effect [18], thus allowing the most flexibility in model development. Several reviews consistently support the fully Bayesian approach over empirical Bayes modelling [16,17,19].

Since there are no closed form analytical solutions for parameter estimates of a fully Bayesian model, nor likelihood profiles to maximize, Markov Chain Monte Carlo (MCMC) methods are used to generate large samples from the posterior distributions of all stochastic nodes of the hierarchical model, given the likelihood describing the original data distribution and all appropriate prior and hyperprior distributions of the likelihood parameters [16]. Estimation and inference in the fully Bayesian paradigm are based upon these large sample approximations of the posterior distributions.

In what follows, the fully Bayesian approach is applied to simulating large samples from the posterior distribution of prostate cancer relative risk in each of 1412 ZIP codes in New York State. Various aspects of these distributions are then mapped to reveal information on the geographic patterns of prostate cancer.

## Results

**Figure 2:**
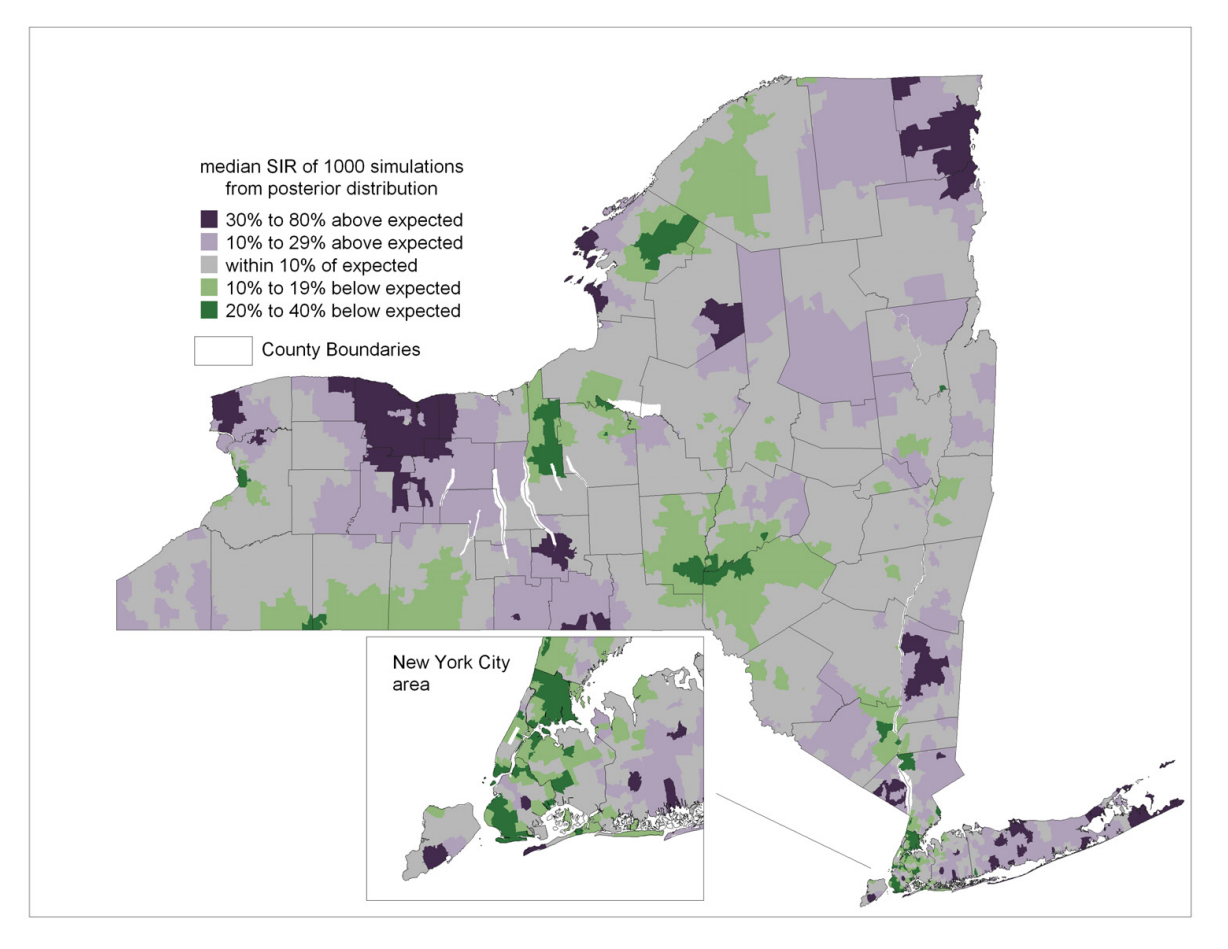
**Bayesian smoothed prostate cancer incidence**. Median of the posterior distribution of ZIP code-level standardized incidence ratios. Thematic categories based on natural breaks method, with slight adjustment.

**Figure 3:**
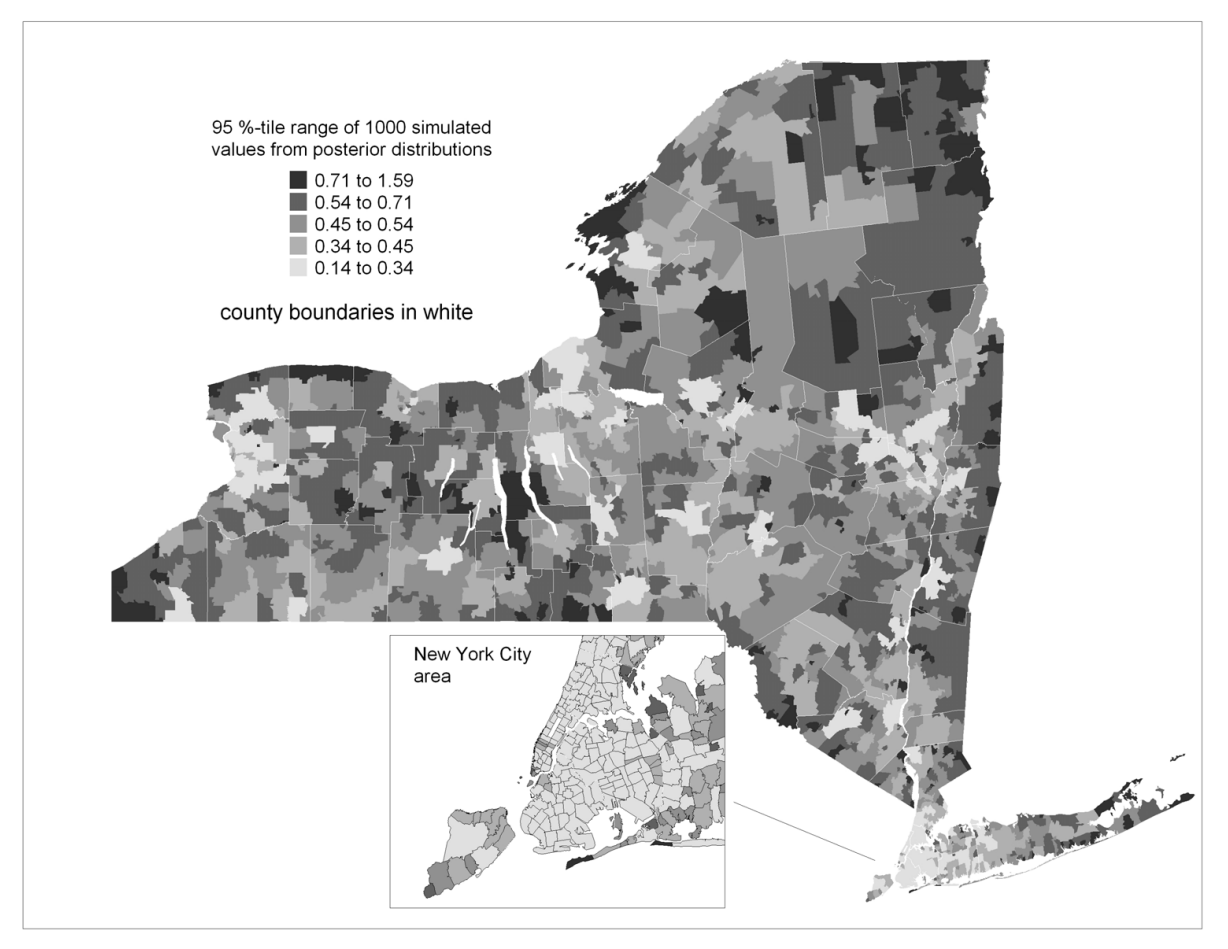
**Uncertainty of Bayesian Smoothed prostate cancer incidence**. ZIP code-level 95^th ^percentile range of posterior distribution of standardized incidence ratios. Thematic categories based on Natural Breaks Method.

The model defined by equations 1–5 was applied to simulate a sample of 1000 independent observations from the stationary posterior distribution of standardized incidence ratios for each ZIP code. Summary statistics and graphical analysis of these empirical distributions indicated that they arose from generally symmetric posterior distributions. Since the sample mean, median and mode were very similar for each ZIP code, the median was chosen to represent central tendency and is mapped in Figure 2. This "smoothed" map of SIRs provides a picture of spatial pattern inherent in the raw data mapped in Figure 1. Uncertainty associated with these estimates of relative risk is mapped in Figure 3 as the 95 percentile range (97.5^th ^– 2.5^th ^percentile) of the 1000 values sampled from the posterior distribution of SIRs for each ZIP code.

**Figure 4:**
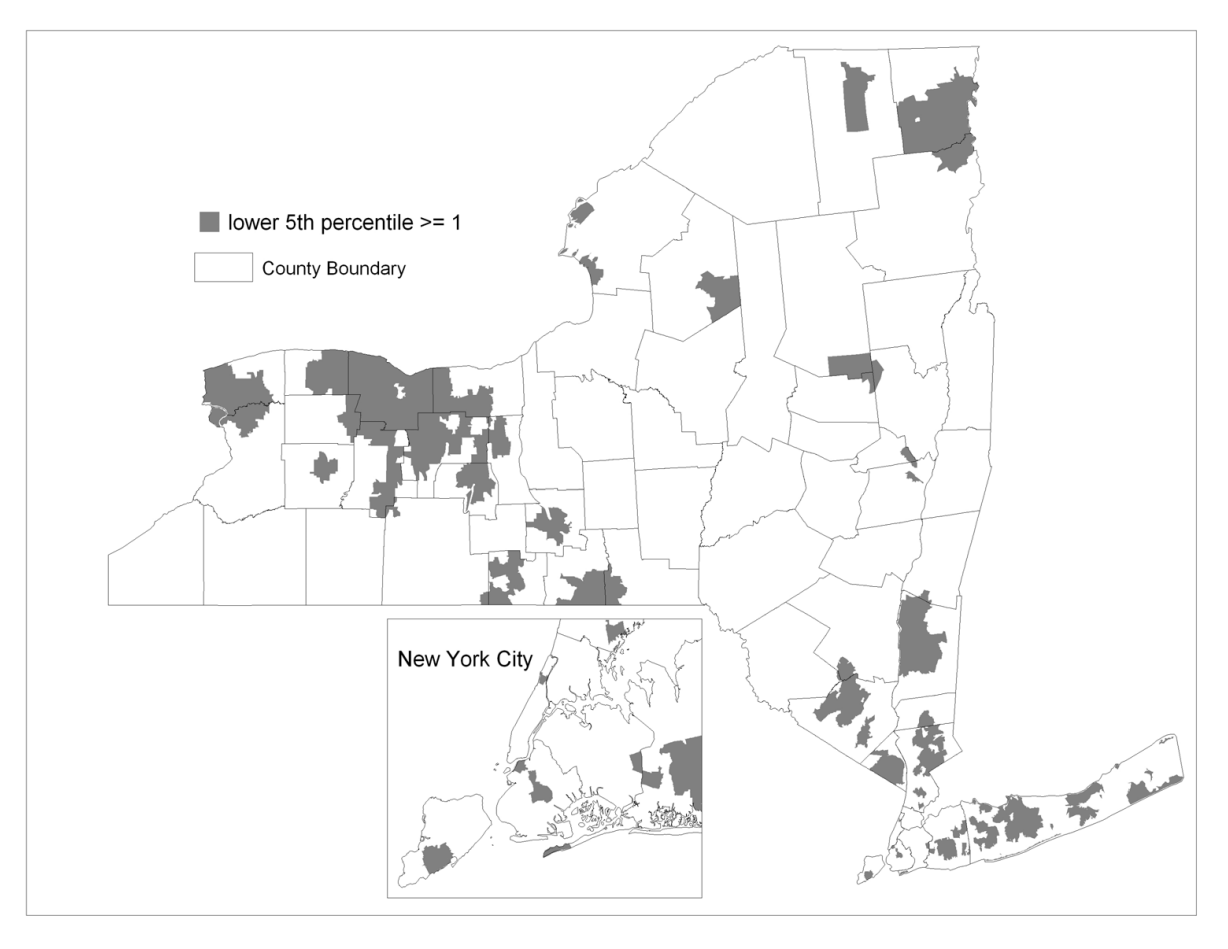
**ZIP codes with a 95% probability of relative risk exceeding 1**. The lower 5^th ^percentile of the posterior distribution of standardized incidence ratios exceeds or equals 1.

**Figure 5:**
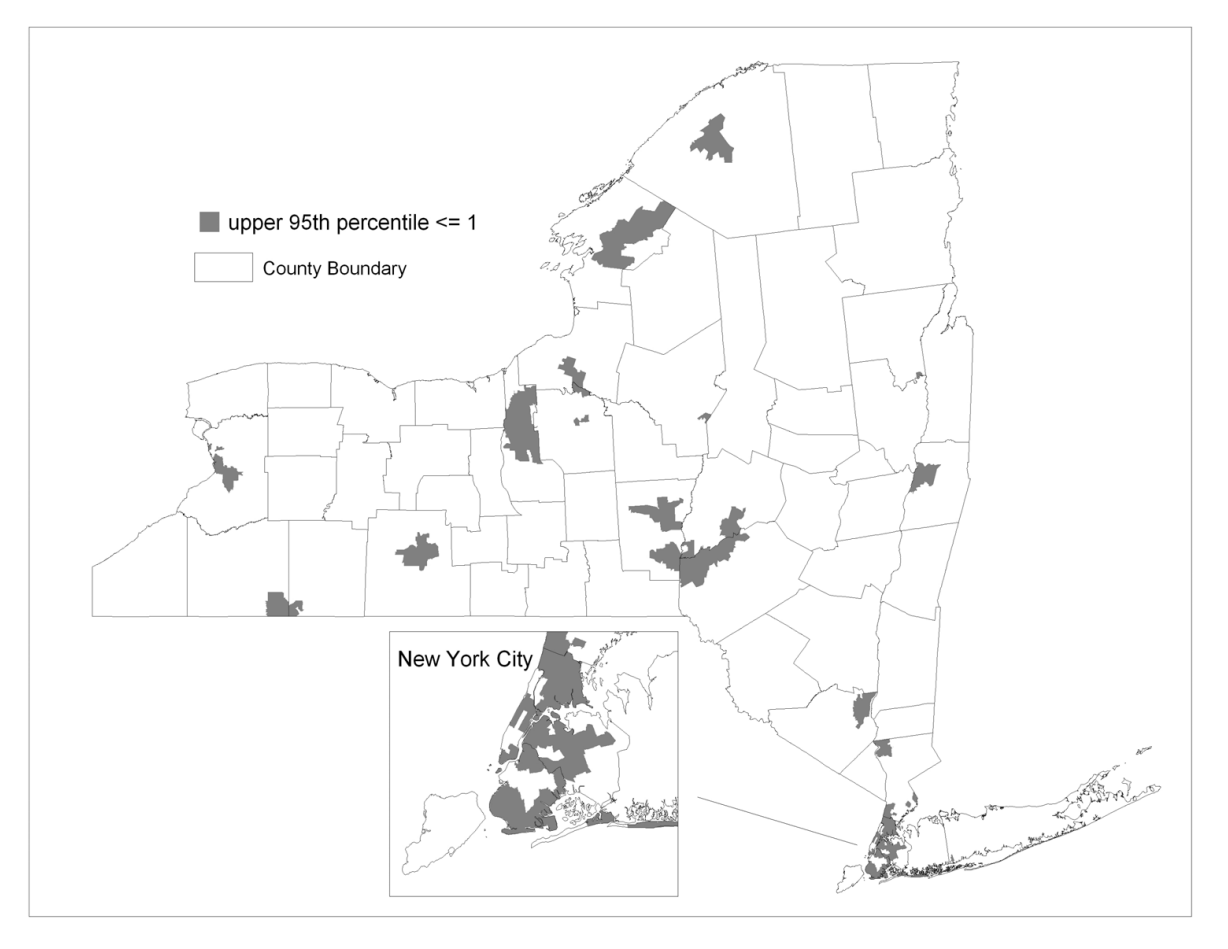
**ZIP codes with a 95% probability of relative risk being less than 1**. The upper 95th percentile of the posterior distribution of standardized incidence ratios is less than or equal to 1.

The posterior distributions can also be used to identify ZIP codes where a specified mass of the distribution of relative risk is greater or less than the null value. For example, Figure 4 shows ZIP codes where 95% of the simulated SIRs exceed the value of one. In the Bayesian paradigm, those ZIP codes highlighted in Figure 4 have a 95% probability of higher than expected risk. Likewise, Figure 5 highlights ZIP codes expressing a 95% probability of lower than expected risk.

## Discussion

### Methodology

The Poisson model applied in this paper is a particular application of hierarchical Bayes spatial generalized linear models for the exponential family of likelihoods [20]. Particular models are specified by the likelihood that is assumed to give rise to the observations, the structure of the prior, and the hyperprior distributions of variance components, which are typically vague to allow learning from the data. An aspect of these models that will influence outcomes is the neighborhood weights, as in Equation (4); however, defining these weights remains an open area of research.

Most applications to date use the first order binary weighting scheme where *w*_*ij *_= 1 if a mapping unit *j *shares a common border with unit *i*, and *w*_*ij *_= 0 otherwise. This weighting scheme actually has its roots in image analysis, for which this type of modelling was developed [14], and it makes sense when the spatial units are equal size and shape pixels and the response variable has a constant variance for each pixel. However, this is not the case when smoothing disease maps where the mapping units are of irregular size and shape, and stability of the response variable estimates varies with changing population size. This problem is especially relevant for mapping units like ZIP codes.

A proper approach may be to define weights as a decay function of geographic distance between population-weighted centroids of the mapping units. This function may be obtained by fitting a model correlogram to residuals that are obtained from a model that does not include a random effect to account for spatial autocorrelation. Cressie and Chan [21] used the empirical variogram of the response variable to determine the range of spatial autocorrelation. For neighborhood distances within this range, weights were defined as a function of Euclidean distance. Meanwhile, Griffith [22] provides some "rules of thumb" for defining geographic weights, but they are very general. Ferrandiz, et al [23] used a weight of *n*_*i*_*n*_*j*_/*d*_*ij *_for neighboring mapping units separated by geographic distance *d*_*ij *_and of population sizes *n*_*i *_and *n*_*j*_. These authors applied such a weight to prostate cancer mortality mapping; however, this gravity-type weighting may be better suited for infectious disease, not chronic disease.

If a decay function is fit from the data, the varying stability of disease rates among the mapping units presents a challenge. Since the Bayesian model is designed to adjust for varying stability, perhaps a hierarchical model like the one applied in this paper can be extended so that the weights in Equation (4) are defined as a decay function whose unknown parameters can be assigned "hyperprior" distributions.

### Application to prostate cancer mapping

Maps like in Figure 1 present a compromise between the need to protect personal privacy and public demand for fine geographic resolution. Such small mapping units are necessary for discerning among communities that can vary drastically across a region with respect to possible risk factors and both population density and demographics. However, this comes with the cost of unstable risk estimates for many mapping units that have small populations. Smoothing is therefore applied to help visualize spatial pattern that is inherent in the data of Figure 1. It is demonstrated how hierarchical Bayes spatial modelling has the appealing feature of providing a whole distribution of possible outcomes that can be used for not only smoothing, but also to explore other aspects of spatial pattern.

Viewing Figures 1 through 3 indicate that the Bayesian model is behaving as expected since the smoothed estimates are increasingly dependent on the prior model as uncertainty increases due to decreasing population, whereas for ZIP codes with large populations, like in New York City, the smoothed estimates are similar to the raw SIRs.

We also see that many edge ZIP codes in less populated areas tend to have greater uncertainty relative to their non-edge neighbors because there are fewer neighbors to borrow strength from. For the heterogeneous Poisson model applied in this paper, Lawson et al [24] suggest treating edge mapping units as a guard and not as part of the actual study area. However, presenting the width of Bayesian posterior distributions provides a way to retain the edge units while also showing the relative uncertainty associated with their smoothed values.

The smoothed pattern in Figure 2 is highlighted for areas of high and low incidence in Figures 4 and 5, respectively. Geographic patterns seen in these maps are potentially influenced by many factors, including differences between regions of the state in terms of racial, ethnic and socio-demographic composition. Yet it is well recognized that interpreting any possible relationships with risk factors is confounded by differences in screening and diagnostic practices across the state. Prostate-specific antigen (PSA) testing remains high among US males over 40 [25] and there is evidence of a steady increase in testing rates in New York State during the years corresponding to the data analyzed in this paper [26]. Along these lines, we note that a relatively large proportion of the four most populated counties of New York City reveal a high probability of less than expected incidence (see Figure 5 inset). This may be partially explained by the large immigrant population in these four counties, as indicated by much higher proportions of people who are foreign-born and/or do not speak English at home [27], which may translate to lower screening rates.

Although the patterns seen in Figures 2, 4 and 5 may be partially explained by geographic variations in PSA testing and diagnostic follow up, such variation is not actually known, therefore we cannot adjust for this potential confounder. In the neighboring state of Connecticut, geographic variation of invasive prostate cancer incidence was large and revealed some consistency before and after the introduction of PSA testing, while the pattern was completely different and variation was much smaller during the years of PSA introduction [28]. These authors suggest that such a space-time pattern reflects the impact of introducing PSA testing, although this cannot be confirmed in the absence of data on geographic differences in PSA use.

Some areas appear elevated that are in popular vacation spots, such as the eastern forks of Long Island and the "north country" of New York State including the Thousand Islands area along the Saint Lawrence River (near Watertown in Figure 1) and the Adirondack region. This may possibly be due to a seasonal residence effect whereby vacation areas tend to have artificially inflated chronic disease rates [29]. This occurs when seasonal residents provide a health care provider with a local address of a vacation home, while their primary residence is where they are counted by the decennial US census. Consequently, if their residence at time of diagnoses is in the vacation area, this record inflates the SIR numerator for that area, while they are counted in the denominator for the area of their primary residence as captured by the census. This effect is enhanced since the population spending extended periods in vacation areas tends to be over age 55, which is the age cohort at highest risk of chronic disease. Boscoe and Mclaughlin [29] have presented evidence of increased overall cancer rates in areas with seasonally resident populations in New York State, especially the Thousand Islands area. This uncertainty is reflected in Figure 3 where the width of the posterior distribution of SIRs for these areas is relatively large.

While the smoothed results in Figure 2 present an advantage over mapping raw data, a limitation of smoothing is that the pattern we decipher is subject to confounding by spatially varying population sizes [30]. In other words, smoothed maps like in Figure 2 reveal high and low relative risk in areas with larger populations, while areas with small populations tend to be smoothed towards the null value. While this means that areas with small populations that actually have abnormally high or low disease rates may be obscured, it is still well recognized that many extreme values associated with small populations may simply reflect random noise.

Other methods like the spatial scan statistic can be used in conjunction with Bayes smoothing to strengthen overall spatial analysis. Statistically significant scan statistic circles like those in Figure 1 can vary in size, potentially encompassing many ZIP codes, so are not restricted to only pre-defined neighborhoods like the conditional autoregressive model used by the Bayes smoothing algorithm. In this regard, the spatial scan statistic is similar to smoothing by spatial filtering with variable-radius circles [10]. General regions of statistically elevated relative risk may be identified by the scan statistic, with supporting evidence from Bayesian posterior distributions to help identify the mapping units that contribute strongly to a scan statistic circle. Indeed, each spatial scan statistic circle reported in Figure 1 contains at least one elevated ZIP code identified in Figure 4 by the Bayesian model.

There is ample flexibility for exploratory analysis by varying the display parameters for results from these two methods. For example, the results in Figure 4 can be either more generalized or further focused by identifying ZIP codes where, say, 90% or 99%, respectively, of the posterior probability mass exceeds the null value of one. At the same time, we can display the set of non-overlapping scan statistic circles that correspond to lower levels of relative risk than are shown in Figure 1, thus capturing more geographic area. In fact, when this is done for scan statistic circles corresponding to 15–49% relative risk (not shown), all of the elevated ZIP codes in Figure 4 are spatially associated with significant scan statistic circles.

There is extensive literature on hierarchical Bayes spatial modelling for disease mapping; however, most papers are theoretical in nature and use illustrative examples, often with the same data sets. One exception was recently published by Short et al [31], who applied Bayes modelling to produce maps of cancer control variables. Specifically, they smoothed maps of different outcomes (mortality, incidence, staging and screening) for each of breast, colorectal and lung cancer in Minnesota (USA) counties. Cancer control maps were created for each cancer site by obtaining a weighted sum of each smoothed outcome, and an overall cancer control map was obtained by a weighted sum of the individual cancer control map values. These results can help guide resource allocation for state cancer prevention and control efforts.

While there are open areas for improvement in the methodology of hierarchical Bayes spatial modelling, it is a valuable tool for geo-spatial assessment of disease patterns that can help identify differences among communities. This may in turn indicate patterns of health care access, screening and diagnostic follow up and possibly indicate etiologic clues about causal relationships.

## Methods

### Data

Observed and expected values of prostate cancer incidence used to calculate SIRs for 1412 New York State ZIP codes [1] were obtained for the years 1994 to 1998 from the New York State Cancer Registry (NYSCR). The expected values used in the SIR denominator are based on indirect standardization using the age-by-race distribution in each ZIP code and the statewide age- and race-specific incidence rates as a reference. Age and race distributions correspond to the year 1997, as estimated by the Claritas Corporation™ based on prior census values. ZIP code boundaries were delineated by the GDT Corporation™ in 1999.

ZIP Code delivery areas are prone to change over time [32], particularly in rapidly growing parts of the country. According to the NYSCR [personal communication], a review of all of the issues of the Postal Bulletin, where these changes are documented, from 1990 to the present revealed that New York has had stable ZIP Code delivery areas. Approximately 50 small, rural post offices were closed, 3 new post offices were added, and none were realigned. ZIP codes were combined in instances where service delivery area changed between 1990 and 1999 or for confidentiality reasons where necessary [33].

### Modelling

Letting the geographic domain (New York State) be subdivided into *i *= 1, ..., *n *distinct mapping units (*n *= 1412 ZIP codes for our application), the number of cases within each unit, *Y*_*i*_, conditional on location *i*, is defined as a Poisson random variable with expectation *E*_*i*_*θ*_*i*_, where *E*_*i *_equals the age- and race-adjusted expected number of prostate cancer cases, and *θ*_*i *_equals the area-specific relative risk. Given an observed response *y*_*i*_, note that the maximum likelihood estimate of relative risk is  = *y*_*i *_/ *E*_*i*_, the standardized incidence ratio (SIR).

The relative risk parameter *θ*_*i *_is assigned a log-normal prior distribution, log(*θ*_*i*_) ~ N(*μ*_*i*_, ), where the expectation and variance are defined by a linear function of a common value (intercept), *α*, and two independent random effects, a heterogeneous component, *u*_*i*_, that does not depend on geographic location (exchangeable) and an autocorrelated component, *v*_*i*_, that reflects local spatial structure by incorporating the influence of neighboring geographic units. Prior distributions are then assigned to these linear terms and consequent hyperprior distributions are assigned to the variance terms, thus creating a 4-level hierarchical model as follows.

Level 1, define the likelihood: *Y*_*i *_~ Poisson(*E*_*i*_*θ*_*i*_)     (1)

Level 2, link to a linear function: log(*θ*_*i*_) = *α *+ *u*_*i *_+ *v*_*i *_    (2)

Level 3, assign prior distributions: *α *~ N(0,0.0001), noting that 0.0001 is the precision, thus defining a vague prior,





for a neighborhood of geographic units *δ*_*i *_with respect to unit *i *and *w*_*ij *_is a weight defining the relationship between geographic unit *i *and its neighbor *j*. The weight is defined simply as *w*_*ij *_= 1 if ZIP codes *i *and *j *are adjacent (share a common border) and *w*_*ij *_= 0 otherwise.

Level 4, assign hyperprior distributions to precision terms:

*τ*_*u *_= 1 /  ~ Gamma(a,b) and *τ*_*v *_= 1 /  ~ Gamma(c,d)     (5)

for shape parameters a and c, and inverse scale parameters b and d.

This is the convolution model originally proposed by Besag, York and Mollie [18], where the random effect associated with spatial autocorrelation, *v*_*i*_, is defined according to the conditional auto-regressive model (CAR) [14]. Note that the distribution of *v*_*i *_is conditional on geographic location, whereby its expectation equals a local neighborhood average. The Bayesian model puts increasing emphasis on this term as the underlying population at location *i *decreases.

Although covariates can be incorporated into the log-linear expression at the second level of the model, our interest is with estimating and mapping the relative risk, *θ*_*i *_= exp(*α *+ *u*_*i *_+ *v*_*i*_).

### Choosing Gamma Hyperpriors

While it is established that a vague prior is acceptable for the linear term *α *in Equation (2) (i.e. Ghosh et al [20]), the model should be evaluated for sensitivity to choice of the Gamma hyperprior distributions of the precision terms, as in Equation (5). Two very different hyperprior specifications that appear in the literature for this convolution model were experimented with. Hyperparameters were specified for one model as Γ(1,1), which yields a probability of 99% that the precision lays between 0.01 and 4.6, and for the other model as Γ(0.5,0.0005), which yields a probability of 99% that the precision lies between 0.16 and 6635, with most of the probability concentrated towards 0. Note that these parameter choices also satisfy sufficient conditions for ensuring a proper joint posterior distribution of all the stochastic nodes [20]. For the statewide collection of New York ZIP code log(SIR)s to be smoothed in this paper, the sample precision equals 6.25 (variance = 0.16) and the precision of first order neighborhood means equals 20 (variance = 0.05). Therefore, it may be desired to retain the model with hyperprior specification of Γ(0.5,0.0005) to at least capture the sample-based precision estimates, while also defining a vague hyperprior that allows more learning from the data.

**Figure 6:**
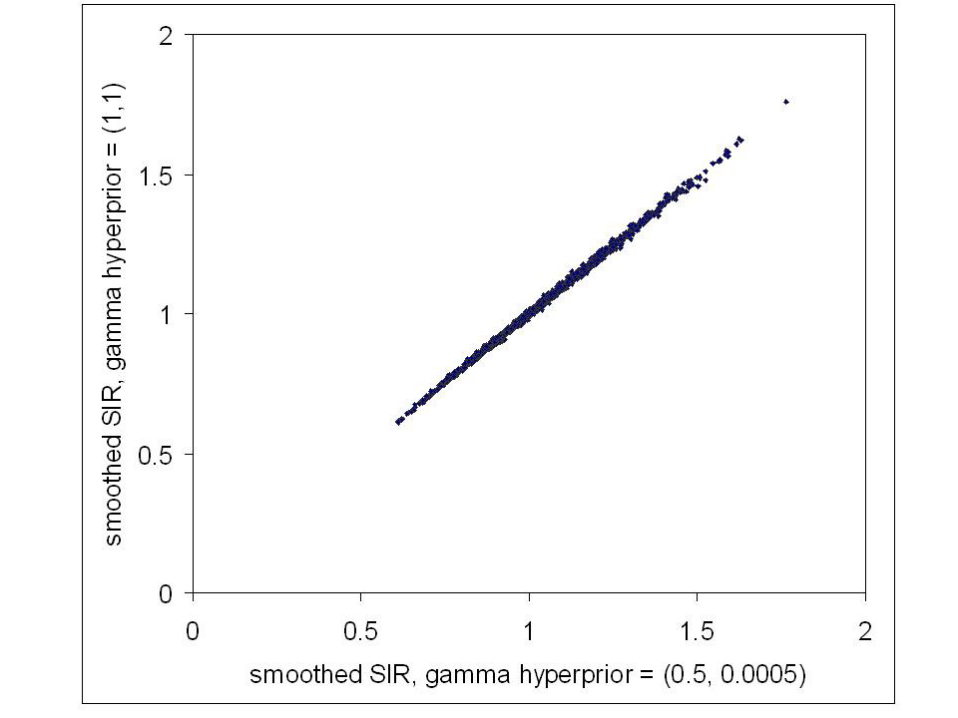
**Comparison of smoothed standardized incidence ratios for two different specifications of the hyperprior distributions**. Each point represents a ZIP code with two medians of the posterior distribution of standardized incidence ratios obtained from different models.

Final smoothed results from each model are compared in Figure 6 where we see, in agreement with Bernardinelli et al [34], that it essentially makes no difference which hyperprior is used. Therefore, the Γ(0.5,0.0005) hyperprior specification was chosen. It is not the intention of this paper to perform a rigorous sensitivity analysis with respect to hyperprior specification; however, the two models assessed in Figure 6 represent very different distributions and therefore indicate that the fully Bayes hierarchical model is quite robust with respect to hyperprior specification when smoothed relative risks are the objective.

### Running the Gibbs Sampler

WINBUGS 1.4 [35] was used for running three independent Markov Chains. Initial values of all stochastic nodes of the model were chosen to provide dispersed initial values without being excessively overdispersed. For the common intercept, *α*, and heterogeneous random effect, *u*_*i*_, zero (0) was used to initiate one chain, plus/minus four standard deviations of the statewide log(SIR) were used to initiate the other respective chains. Zero is the statewide average log(SIR) that provides a point estimate for *α*, plus it is the expected value of *u*_*i*_. For the random effect associated with local spatial clustering, *v*_*i*_, the initial values were based on the average plus/minus four standard deviations of the log of first order neighborhood average SIRs. For the precision terms *τ*_*u *_and *τ*_*v*_, the inverse of the sample variances of the log(SIR) and the log of the first order neighborhood average SIRs were used respectively for one chain, plus lower and higher values were chosen for the other two chains in order to be well dispersed from the middle value, but not wildly so with respect to what is reasonably expected based on the observed spatial variability.

Convergence of relative risk for the three independent chains was confirmed by graphing their traces and observing random mixing of all chains, which revealed white noise variation around a common value, with no trend. This was supported by observing Brooks-Gelman-Rubin diagnostics that clearly satisfied convergence criteria [36]. After a burn-in of 10,000 iterations, which was far more than actually necessary, the following 1000 iterations were sampled from each of the three chains by choosing every third iteration to help avoid possible autocorrelation within a chain. This large sample approximation of the stationary posterior distribution for each ZIP code relative risk was then summarized in WINBUGS and brought into a Geographic Information System [37] for mapping.

### Model Selection

Variations of the model defined above were compared by evaluating the mean deviance of 1000 iterations chosen from the three independent Markov Chains after burn-in. This was done by obtaining the mean of -2(log likelihood) for each iteration, as provided by the *deviance *node in WINBUGS. The mean deviance was then calculated as *D*(**y**, *μ*) = 2 [*l*(**y**, **y**) - *l*(**y**, *μ*)], where *l*(**y**, **y**) is the mean maximum achievable log likelihood, obtained for a saturated model where a parameter is assigned to each datum, and *l*(**y**, *μ*) is the mean log likelihood obtained for the model in question. This takes the conventional assessment of deviance for generalized linear models [12] and applies it to the many outcomes of Monte Carlo simulation, as per Spiegelhalter et al [38].

Incorporating a random effect associated with local spatial structure (CAR term) provides much stronger prior information than the exchangeable random effect alone (Table 1), which assumes purely heterogeneous variation across the state. This agrees with findings by Spiegelhalter et al [38], who developed the Deviance Information Criterion as a penalized version of deviance and applied it to Scottish lip cancer data. The convolution model was therefore chosen, which incorporates both random effects.

**Table 1:** Deviance analysis. See text for explanation.

*Model*	*Mean (-2 LL)*	*Mean Deviance*
Saturated	8082.0	
Convolution	8194.0	112.0
CAR only	8191.0	109.0
Exchangeable only	10800.0	2718.0
